# Entropy in Nanofluids

**DOI:** 10.3390/e20050339

**Published:** 2018-05-03

**Authors:** Giulio Lorenzini, Omid Mahian

**Affiliations:** 1 Department of Engineering and Architecture, University of Parma, Parco Area delle Scienze 181/A, Parma 43124, Italy; 2 Center for Advanced Technologies, Ferdowsi University of Mashhad, Mashhad 91775-1111, Iran; 3 School of Aeronautic Science and Engineering, Beihang University, Beijing 100191, China

**Keywords:** Nanofluids, Entropy generation, Heat Transfer

The study on entropy generation and exergy analysis in Nanofluid flows started in 2010 [1]. After this date, many other studies have been done on this topic such as [2,3,4,5,6]. Figure 1 shows the increase in the number of research and review articles in the field of entropy generation in nanofluid flows. The data are obtained from Scopus, by searching the keywords of “Nanofluids” or “Nanofluid” and “Entropy Generation” or “Exergy”. As shown, the interest to this subject has increased year by year so that in 2017 the number of articles reached more than 100. 

Because of high interest toward this area, in 2016 and 2017 we decided to serve as guest editors for two special issues on nanofluids. The total number of articles that have been published in the two special issues are 21 articles with more than 200 citations. The high number of citations shows the significance of this topic for researchers. 

Here, we give a summary of papers published in these two issues. In the first paper, Kolsi et al. [7] studied three-dimensional entropy generation due to natural convection in a cavity where a diamond-shaped body was installed in the middle of cavity. They used Al_2_O_3_–water nanofluid as the working fluid. It was found that total entropy generation increases with the increasing volume fraction of nanoparticles. In References [8,9,10,11,12], entropy generation due to nanofluid flow on stretching/shrinking surfaces has been studied at different conditions. Freidoonimehr et al. [13] modeled the magneto-hydrodynamics nanofluid flow over a porous disk by considering soret and dufour effects through the Homotopy Analysis Method. 

Li et al. [14] studied the entropy generation of Al_2_O_3_–water nanofluids in a microchannel with flow control devices including cylinder, rectangle, protrusion, and v-groove. They concluded that protrusion devices are the best option to achieve minimum entropy generation. In another work, Xie et al. [15] studied the effect of nanofluid on the entropy generation in rectangular channels with dimples and protrusions. It was encouraged to use dimples and protrusions in the channel since these reduce entropy generation. They showed that using nanoparticles, especially at low Reynolds numbers, leads to decreases in entropy generation. 

Rashidi et al. [16] studied the entropy generation of MHD blood flow of nanofluid due to peristaltic waves. Abbas [17] investigated analytically the entropy generation in the flow of peristaltic nanofluids in channels with compliant walls. Chamkha et al. [18] examined the entropy generation due to natural convection of Cu–water nanofluids in C-shaped cavity where a magnetic force is applied to the flow. Selimefendigil [19] simulated the entropy generation in an entrapped trapezoidal cavity with MHD flow using Al_2_O_3_–water nanofluid. Sheremet et al. [20] evaluated the entropy generation in a square cavity where a solid body was installed inside it using nanofluid. Nasiri et al. [21] studied the flow of Fe_3_O_4_–water nanofluid in a microchannel heat sink with offset fan-shaped reentrant cavities. 

Qasim et al. [22] simulated the entropy generation due to methanol-based nanofluid flow in a sinusoidal wavy channel. Bhatti et al. [23] determined the entropy generation on electro-kinetically modulated peristaltic propulsion in a microchannel using nanofluids by considering joule heating. Baskaya et al. [24] presented the entropy generation in an inclined channel under a magnetic field where a ferrofluid (Cu–water) fills the channel. Sheremet et al. [25] solved the entropy generation due to natural convection flow of nanofluids in a cavity with non-uniform distribution of temperature on the left wall by considering the effects of Brownian motion and thermophoresis. In the last work, Al-Rashed et al. [26] studied the entropy generation due to 3D natural convection flow of Carbon Nanotubes(CNTs)–water nanofluids in a square cavity where a baffle with arbitrary velocity could rotate inside it.

In most of the studies mentioned above, it was concluded that adding nanoparticles leads to heat transfer enhancement, and on the other hand, entropy generation reduces with an increase in the volume concentration of nanoparticles.

It is suggested for future studies that authors:

1. Investigate the effects of different thermophysical models (especially correlations developed based on experimental data) on entropy generation rate;
2. Use both two-phase mixture model and single-phase models and compare the results;
3. Investigate new configurations;
4. Investigate entropy generation in new application of nanofluids, as most of the present studies are limited to classic problems such as flow on sheets or inside cavities and ducts;
5. Consider the prediction of entropy generation using soft computing approaches like neural network;
6. Conduct a comparison between entropy generation rates of different nanoparticles and base fluids to recognize the optimum nanofluid from the second law of thermodynamics viewpoint.

## Figures and Tables

**Figure 1:**
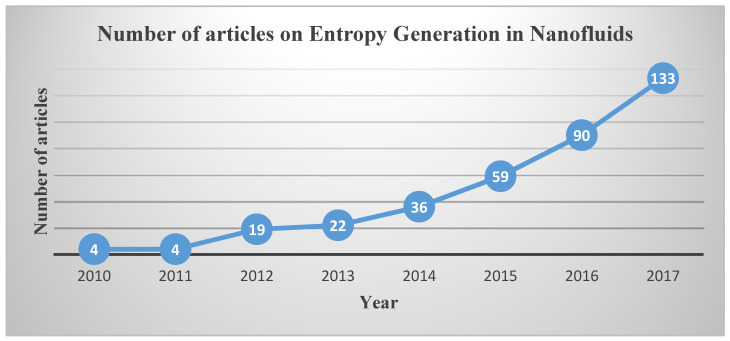
Number of articles on entropy generation in nanofluid flow.
